# Supplementary material for: Isolation of Neoantigen-Specific Human T Cell Receptors from Different Human and Murine Repertoires
Source: Cancers (Basel). 2022 Apr 6;14(7):1842. doi: 10.3390/cancers14071842 (PMC8998067; doi:10.3390/cancers14071842)
Supplement: Supplementary file 1 [file cancers-14-01842-s001.zip › Supplementary Figures.pdf]

Supplementary Figures

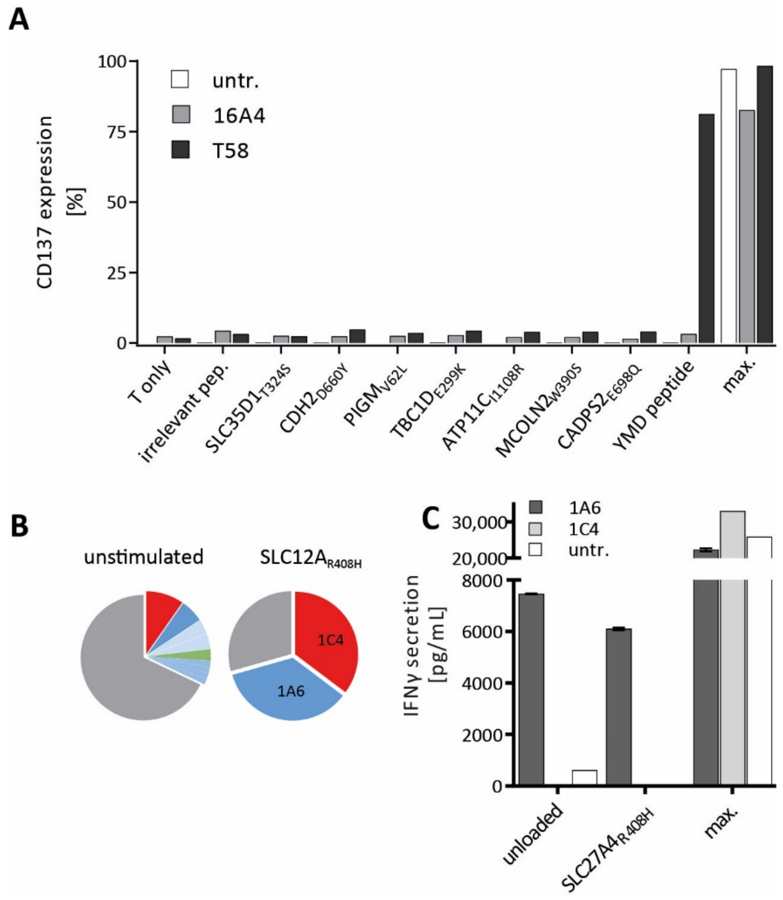

Figure S1. Test of patient derived TCRs from single-cell sorting from patients BIH146 and BIH56. (**A**) Peptide recognition assay of peptide-loaded target cells by TCR 16A4-transduced (16A4-td) PBLs identified for patient BIH146. Peptide reactivity was measured by surface expression of activation marker CD137 on 16A4-td PBLs after a 20h co-culture with peptide-loaded target cells. Activation marker expression from one donor is shown. Alive, single cells were gated on CD8+, mTCRß+. (**B**) Single-cell sequencing of peptide-stimulated PBMCs of patient BIH56. Pie charts show frequencies of detected TCR clones of unstimulated and neoepitope candidate SLC12A$_{R408H}$ stimulated T cells. TCRs of 1C4 and 1A6 were identified, grey segments represent all TCRα/β rearrangements which were detected only once per sample. (**C**) TCR 1C4 and 1A6 were expressed on PBLs and tested for reactivity in a co-culture with peptide-loaded target cells. IFNγ-release was measured after an overnight co-culture in duplicates, error bars show standard deviation. Untr.: untransduced; T58: control TCR specific for Tyrosinase peptide (YMD: YMDGTMSQV); max.: PMA/Ionomycin for maximal; unspecific activation.

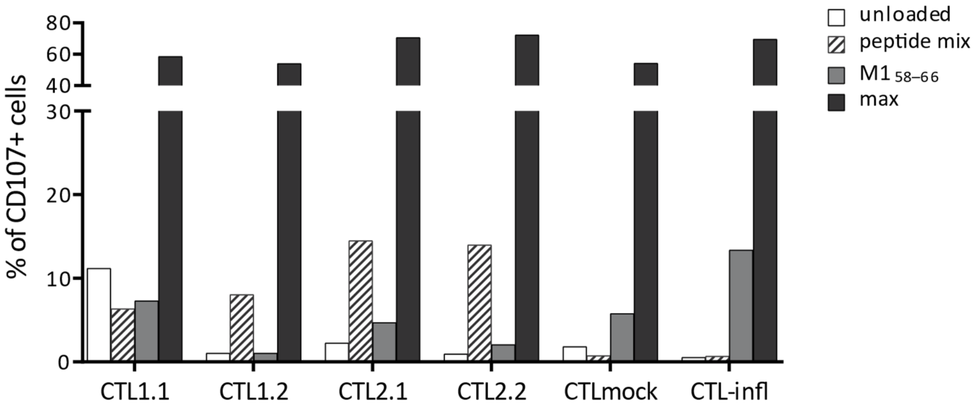

Figure S2. Degranulation assay of cytotoxic T cell lines (CTLs). CTLs were generated by stimulation of bulk CD8+ T cells from healthy donor A with a peptide pool of three candidate neoantigen peptides ($CDH2_{D660Y}$, $SLC35D1_{T324S}$ and $PIGM_{V62L}$). Peptide reactivity was assessed by measuring expression of degranulation marker CD107 after incubation with peptide-loaded target cells by flow cytometry. The surface expression of CD107 on CTLs is shown for 4 generated CTL lines from donor A. CTLs generated against influenza peptide $M1_{58\text{-}66}$ were used as an assay control. CTL were gated on single, viable, CD8+ cells.

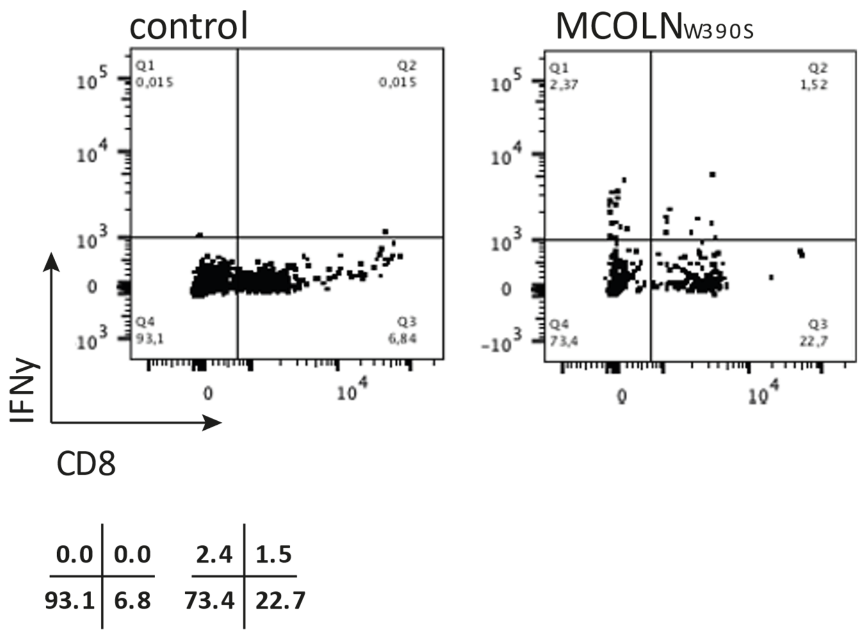

Figure S3. Generation of neoepitope candidate specific TCRs in ABabDII mice for patient BIH146. Splenocytes of immunized ABabDII mice were cultured to expand peptide reactive CD8+ cells. Expanded cells were sorted after an IFNγ capture assay and IFNγ+ cells were sorted. Cells were gated on CD3+CD8+IFNγ+ cells. Cells cultured without peptide served as a negative control. Plots are shown for peptide $SLC12A_{R408H}$ stimulated splenocytes, from which TCR m875 was identified.

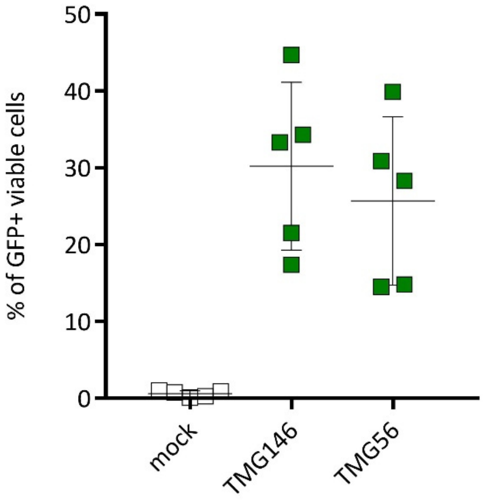

Figure S4: Nucleofection of multiple myeloma cell line U266 with TMG-constructs. U266 cells were nucleofected with neoepitope-encoding Tandem Minigenes (TMG). Nucleofection efficiency was determined 16-20 h post nucleofection by detection of eGFP expression via flow-cytometry of viable cells (n=5). Mock cells were nucleofected with PBS. Cells were immediately used as target cells in co-culture experiments. TMG146 – TMG encoding for predicted neoepitope candidates for patient BIH146; TMG56 - TMG encoding for predicted neoepitope candidates for patient BIH56.
